# Effects of methylphenidate on motor system excitability in a response inhibition task

**DOI:** 10.1186/1744-9081-5-12

**Published:** 2009-02-27

**Authors:** Oliver Kratz, Martin S Diruf, Petra Studer, Wolfgang Gierow, Johannes Buchmann, Gunther H Moll, Hartmut Heinrich

**Affiliations:** 1 Department of Child and Adolescent Psychiatry, University of Erlangen-Nürnberg, Schwabachanlage 6, 91054 Erlangen, Germany; 2 Heckscher-Klinikum München, Deisenhofener Strasse 28, 81539 München, Germany; 3 Department of Child and Adolescence Psychiatry and Neurology, Center of Nerve Diseases, University of Rostock, Gehlsheimer Strasse 20, 18147 Rostock, Germany

## Abstract

**Background:**

Motor system excitability is based on a complex interaction of excitatory and inhibitory processes, which in turn are modulated by internal (e.g., volitional inhibition) and external (e.g., drugs) factors. A well proven tool to investigate motor system excitability in vivo is the transcranial magnetic stimulation (TMS). In this study, we used TMS to investigate the effects of methylphenidate (MPH) on the temporal dynamics of motor system excitability during a go/nogo task.

**Methods:**

Using a double-blind, placebo-controlled, crossover design, 14 healthy adults (8 male, 6 female; aged 20–40 yrs) performed a spatial go/nogo task (S1-S2 paradigm) either under dl-methylphenidate (MPH, 20 mg) or placebo. TMS single and double-pulses (interstimulus interval: 3 ms) were delivered either at 120, 230 or 350 ms after the S2 stimulus (control, go and nogo trials).

**Results:**

At the performance level, faster reaction times and a trend towards less impulsivity errors under MPH vs. placebo were observed.

In nogo trials, i.e., when a prepared response had to be inhibited, motor evoked potentials (MEPs) had a smaller amplitude at an interval of 230 ms compared to 120 and 350 ms. The short-interval intracortical inhibition (SICI) increased over time.

Under MPH, SICI in nogo trials was larger compared to placebo. With the interval between S2 and the TMS-pulse increasing, MEP amplitudes increased under MPH in nogo trials but an early inhibitory effect (at 120 ms) could also be observed.

**Conclusion:**

Our results show a distinct pattern of excitatory and inhibitory phenomena in a go/nogo task. MPH appears to significantly alter the dynamics of motor system excitability. Our findings suggest that a single dose of 20 mg MPH provides some fine-tuning of the motor system in healthy adults.

## Background

Motor system excitability is based on a complex interaction of excitatory and inhibitory processes [1]. Over the last two decades, transcranial magnetic stimulation (TMS) has proven to be an appropriate tool to study motor system excitability in neurological disorders [2] and psychiatric disorders [3,4] but also to study the effects of CNS active drugs [5].

Among the TMS procedures is the double-pulse paradigm first described by Kujirai et al. [6]: The basic principle involves the application of two TMS stimuli via the same coil. The intensity of the first stimulus is set below and the second one above the motor threshold (MT). If the interstimulus interval (ISI) is between 6 and 25 ms, intracortical facilitation (ICF) occurs, i.e., the MEP amplitude measured at the target muscle is larger than for a single supra-threshold stimulus. If the ISI is set to 1–5 ms, the opposite effect emerges. The MEP response is inhibited, so-called short-interval intracortical inhibition (SICI) occurs. If both pulses are above the MT and the ISI is between 50 and 200 ms, two MEPs are elicited, with the second one of a smaller amplitude (long-interval intracortical inhibition, LICI) [7].

Different interneuronal networks in the motor cortex account for these intracortical excitability phenomena which are affected differentially by neuromodulators (e.g., dopamine, noradrenaline) [1,5].

In children with attention-deficit hyperactivity disorder (ADHD), a decrease in SICI was reported in several studies [8-10] probably reflecting a neurophysiological correlate of motor hyperactivity and an inhibitory deficit in these children, respectively. In adult patients with ADHD, a reduced SICI was also found [11,12]. Methylphenidate (MPH), a dopamine-/noradrenaline reuptake inhibitor, which is considered as first-line treatment, enhances SICI in children with ADHD [8] with effects being correlated with clinical improvements [10].

For healthy adults, opposite effects of a single dose of MPH (10 mg) on intracortical excitability were described [13]. ICF was enhanced with SICI being unchanged. However, dosage or genetic factory may influence the results [14-16].

Motor system excitability cannot only be studied with subjects at rest, but also while performing a motor control task [1]. Motor (movement) control is associated with different excitatory and inhibitory effects so that it may be compared with a car with gas and brake pedals [17]. In a reaction task for example, excitability in the area projecting to the agonist muscle increased just before the movement as reflected by larger amplitudes to TMS single-pulse responses. Showing a different time course, the SICI decreased continuously before the start of EMG activity, from 60 ms before EMG onset, even facilitation is possible [18,19]. Leocani et al. described a bilateral reduction of MEP amplitudes to single pulses in nogo trials of a go/nogo task at a time corresponding to the mean reaction time in go trials. Since this inhibitory effect also occurred on the side not to be moved, this finding indicates that suppression of movement is an active process [20].

Sohn et al. demonstrated that SICI increased under volitional inhibition in a go/nogo task [21]. Thus, SICI may play a role in providing nonselective suppression of voluntary movement in addition to focusing the subsequent excitatory drive to produce the intended movement.

MPH may affect the interplay of excitatory and inhibitory processes during a response inhibition task. The aim of this study was to address this issue in healthy adults, using a double-blind, placebo controlled, crossover design. We were specifically interested how overall excitability and SICI develop over time (350 ms poststimulus) when a prepared response has to be inhibited and how these temporal dynamics are modulated by MPH.

## Methods

### Subjects

14 subjects (8 male, 6 female; aged 20–40 yrs) without neurological or psychiatric impairments, psychotropic medication and cardiac arrhythmia took part in our study. 12 subjects were right-handed, two males were left-handed. The study was conducted in accordance with the Declaration of Helsinki. All subjects gave their written informed consent to participate in the study, which was approved by the Ethics Committee of the University of Erlangen-Nürnberg.

### Procedure

The study drug (dl-methylphenidate, MPH, 20 mg or placebo) was administered orally in a randomized, balanced order. The two measurements were done at the same time of day one week apart. 60 min after intake of either placebo or MPH, the TMS resting motor threshold was determined. The go/nogo task started 70 min after intake of medication and lasted for about 50 min.

**Figure 1:**
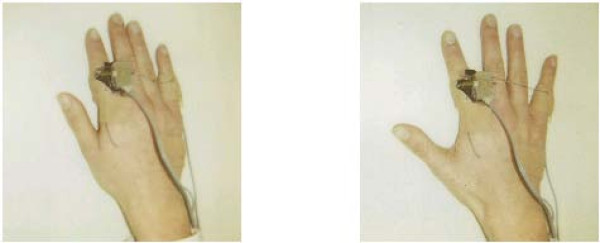
**Device to register reactions**. Device to register reactions (spreading of the hand with the m. abductor digiti minimi involved). left side: switch open, right side: switch closed.

Subjects were seated 100 cm in front of a 17" monitor connected with a personal computer. As input device an apparatus was used that recognized spreading of the fingers via a plastic loop connected with a switch (see Fig. 1) and transmitted the data via a circuit board of a customary usb-keyboard (analogous pressing keys "r" for the right hand, respectively "l" for the left one).

### TMS

During the experiment single- and paired-pulse TMS was applied using a figure-of-eight coil (diameter of one wing = 70 mm) connected to a Magstim^® ^Bistim unit with two Magstim^® ^200^2 ^stimulators (Magstim, Whitland, UK).

The optimal stimulation position was determined over the left motor cortex to elicit motor evoked potentials (MEPs) of the m. abductor digiti minimi of the right hand. Resting motor threshold (RMT) was defined as the minimal stimulus intensity that did not elicit a motor evoked potential of more than 50 μV in five consecutive trials in the resting target muscle. The inter-stimulus-interval (ISI) of the paired pulses was set to 3 ms.

The suprathreshold stimulus intensity was adjusted (10–15% above resting motor threshold) so that the MEP amplitude was about 1 mV (peak-to-peak), the conditioning stimulus intensity was set to 75% of RMT.

### EMG recording

EMG recordings were made from the abductor digiti minimi muscle of both hands with the recording of the left hand serving as artefact control. Ensuring a comfortable posture to minimize EMG artefacts, both forearms were supported by armrests. EEG was recorded simultaneously but these data will not be reported here.

A Brainamp^® ^recording system (standard Brainamp amplifier; Brainproducts, Gilching, Germany) was used for data acquisition (bandwidth: 8–1000 Hz, sampling frequency: 5 kHz).

### Go/nogo task and TMS conditions

**Figure 2:**
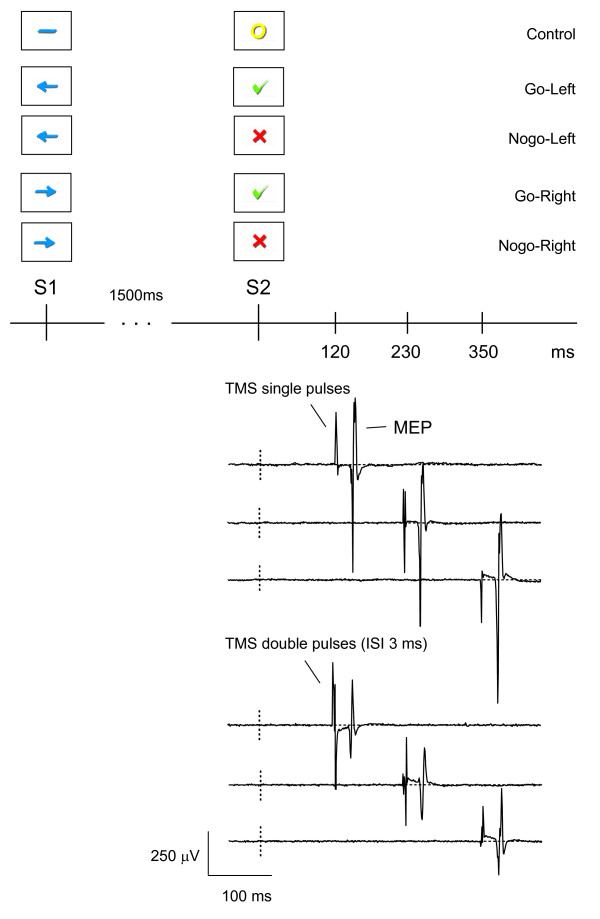
**Illustration of the go/nogo task (S1-S2 paradigm)**. The go/nogo task consisted of control, go-left, nogo-left, go-right and nogo-right trials. TMS stimuli (single pulses or double pulses with an interstimulus interval of 3 ms) were presented either at 120 ms, 230 ms or 350 ms after the onset of the S2 stimulus. The EMG traces, which were recorded during control trials, contain the TMS stimulus artefacts and the motor evoked potentials (MEP) elicited by the TMS stimulus.

Presentation^® ^(Version 11.0; Neurobehavioral Systems, Albany, CA, USA) was used for the presentation of the go/nogo-task and for the synchronized triggering of the magnetic stimulators. The task consisted of five experimental blocks with 60 trials per block. Each trial started with the presentation of a cue stimulus ("S1", 250 ms duration), which was followed, after an interval of 1500 ms, by a second stimulus ("S2", 250 ms duration; see Fig. 2). The intertrial interval (S1 – S1) was 5500 ± 500 ms. S1 was either an arrow pointing to the left, one pointing to the right or – as a control stimulus- a horizontal line. S2 was either a green check (go-signal), a red cross (nogo-signal) or a yellow circle (control-condition). The direction of the arrows instructed the participant which hand to spread in reaction to a go-signal coming up as S2. Thus, there were five different task trials (control, go-left, go-right, nogo-left, nogo-right; equal probability).

TMS was always applied over the left motor cortex and MEPs were always recorded at the m. abductor digiti minimi of the right hand. In the terms "go-left", "go-right" etc. side determines the hand associated with a task response.

To introduce some time pressure and to enhance the task difficulty, respectively, participants received a monetary reward (6 cent per trial) for correct responses in the go trials which occurred within 350 ms after the S2 stimulus. In case of a wrong reaction, the same amount of money was subtracted.

To get familiar with the task including the TMS stimulus which could interfere with the task completion, short practice blocks were introduced. In four of the five experimental blocks TMS was applied. The block without TMS varied randomly between the second and the fourth block.

In each trial of the TMS blocks, either a single-pulse or a double-pulse TMS stimulus was applied. TMS stimuli occurred at a latency of 120, 230 or 350 ms after the S2 stimulus. So, there were 30 different task × TMS conditions. In each block, there were two trials of a task × TMS condition (e.g., nogo-left trial × TMS single pulse at a latency of 230 ms). The order of the different task × TMS conditions varied randomly across task blocks.

### Data processing and analysis

The recorded EMG data were subdivided into segments with one segment per trial. Only trials with correct behavioral response were processed. For each trial, two peak detections were processed: One, within a window of 100 ms duration, starting 100 ms before the magnetic pulse to detect initial tension of the muscle of either hand. Trials with an initial EMG activity larger than 50 μV were excluded. The second peak detection was processed to determine the maximum MEP amplitude (peak-to-peak). MEP amplitudes larger than 3000 μV and smaller than 50 μV were also not considered for further analysis. So far, there is no standard procedure how to analyze TMS data which are recorded during a response inhibition task. If intracortical excitability is measured at rest, only sub-blocks with unconditioned MEPs in the range of 500–1500 μV are usually considered [e.g., [10]]. For the analysis of response inhibition tasks, a different strategy has to be applied. We decided to use the upper and lower limits mentioned above in order to eliminate extreme values which are rather due to artefacts or technical reasons.

The mean amplitude for each task × TMS condition was calculated. Since MEP amplitudes of two different measurements were compared, absolute MEP amplitudes can hardly be used. Therefore, relative MEP amplitudes were calculated with the arithmetic mean of the single pulse MEPs of the particular control condition being defined as 100 percent.

In go-right trials, mean reaction time was about 320 ms (see Table 1). EMG activity started about 90 ms earlier before a reaction was triggered and registered. Also taking the MEP latency of about 20 ms after the TMS stimulus into account, it is obvious that EMG activity was present in most of the go-right trials when TMS was applied 230 ms or 350 ms after S2. So, go-right trials with these TMS latencies were not further analyzed.

**Table 1:** Performance results.

	**Placebo** **Mean ± SD**	**MPH** ** Mean ± SD**	**ANOVAs (significant results)**
Hit rate (in %)	98.1 ± 1.8	98.6 ± 1.5	
Impulsivity errors	1.07 ± 1.14	0.57 ± 0.65	Med: F(1,12) = 3.27; p < 0.1
Mean reaction time			
left hand (in ms)	335.3 ± 16.5	327.4 ± 16.2	Med: F(1,12) = 4.71; p < 0.05 Med × Order: F(1,12) = 10.43; p < 0.01
right hand (in ms)	320.2 ± 15.0	313.4 ± 15.9	Side: F(1,12) = 27.26; p < 0.001
Reaction time – standard deviation			
left hand (in ms)	39.3 ± 6.5	36.1 ± 7.2	
right hand (in ms)	38.8 ± 5.8	37.6 ± 10.0	

Hit rate, impulsivity errors and reaction times were examined.

Factors in the ANOVAs were type of medication (placebo vs. MPH, 'Med'), the order of medication (MPH at the first vs. the second testing, 'Order') and the reacting hand ('Side').

### Statistical analysis

Data are presented as means ± SD in the text.

Hits (correct responses in go trials) and impulsivity errors (responses in nogo trials) were subjected to an ANOVA with the order of drug administration as between-subject factor and medication (Med, placebo vs. MPH) as within-subject factor. To differentiate between left-hand and right-hand responses, an additional within-subject factor side was considered for the analysis of mean reaction times and the standard deviation of reaction times. Mean reaction time of the blocks with TMS were compared to the block without TMS in order to check if performance was affected by the TMS stimulus.

According to previous TMS studies dealing with response inhibition tasks [19-21], control, go and nogo trials were analyzed separately. For the control and go-left condition, normalized MEP amplitudes were subjected to an ANOVA with one between-subject factor 'Order' and three within-subject factors 'Medication' (Med, placebo vs. MPH), 'Latency' (120 ms, 230 ms, 350 ms) and 'Pulse' (single vs. double-pulse stimulation).

The main focus of the analysis was on nogo trials for which an additional within-subject factor 'Side' (left vs. right) was introduced. Since we were particularly interested in the temporal development of the MEP amplitudes, we conducted trend analyses in case of significant effects containing the factor latency. Thus, it could be determined whether the TMS measures show a linear or a quadratic behaviour from 120 ms, 230 ms to 350 ms. For simplicity, only trend scores are reported here.

In case of a significant interaction effect containing the factor Pulse, we additionally calculated ANOVAs with the standard SICI measure (ratio of conditioned and unconditioned MEP response).

Degrees of freedom were adjusted with Greenhouse Geisser correction where appropriate. SPSS for Windows (version 16.0) was used for statistical analysis.

## Results

### Performance measures

All participants coped well with the go/nogo-task. Performance results are summarized in Table 1.

Hit rate did not differ significantly between MPH and placebo condition (MPH: 98.6 ± 1.5%; placebo: 98.1 ± 1.8%, factor Med: F(1, 12) = 1.71, n.s.).

Impulsivity errors, i.e., reacting despite of a nogo-stimulus, were low but a trend for fewer impulsivity errors under MPH was obtained (MPH: 1.07 ± 1.14, placebo: 0.57 ± 1.65; factor Med: F(1,12) = 3.27, p < 0.1).

Under MPH, subjects reacted slightly, but significantly faster in go trials (MPH: 320.4 ± 11.7 ms; placebo: 327.8 ± 13.5 ms; factor Med: F(1,12) = 4.71, p < 0.05). We obtained a significant Med × Order effect indicating that subjects had shorter reaction times when performing the task for the second time (first testing: 329.3 ± 12.0 ms, second testing: 319.4 ± 15.6 ms; F(1,12) = 10.43, p < 0.01). Right-hand responses were significantly shorter than left-hand responses (left-hand reactions: 331.4 ± 14.0 ms; right-hand reactions: 317.4 ± 11.7 ms; factor Side: F(1,12) = 27.26, p < 0.001). For the standard deviation of reaction times, no significant effect concerning medication was found (F(1,12) = 2.15, n.s.).

Mean reaction time in the task block without TMS was not significantly different compared to the blocks were TMS was applied (block w/o TMS: 324.8 ± 5.0 ms; blocks with TMS: 324.3 ± 4.5 ms; F(1,12) = 0.05, n.s.).

### TMS measures

Data from two subjects had to be excluded because too many trials were rejected by the artifact procedure.

No significant differences between MPH and placebo were found for the resting motor threshold (MPH: 45.6 ± 8.7% of maximum stimulator output, placebo: 45.7 ± 6.6%; F(1,10) = 0.01, n.s.) and the mean MEP amplitude in control trials (MPH: 1.04 ± 0.50 mV, placebo: 0.98 ± 0.44 mV; F(1,10) = 0.07, n.s.).

**Figure 3:**
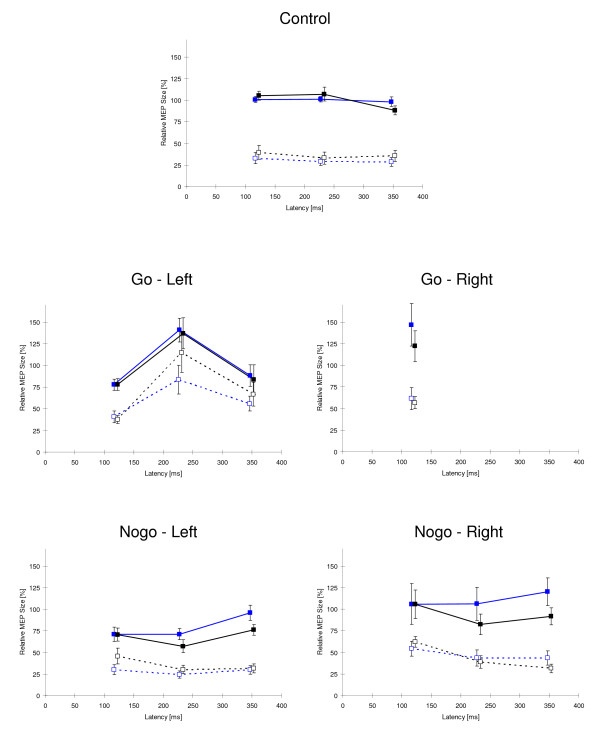
**Relative MEP amplitudes in control, go and nogo conditions**. Relative MEP amplitudes ± SE in control (top), go (middle) and nogo conditions (bottom). The interval between the S2 stimulus and the TMS pulse in milliseconds is plotted against the x-axis ("latency"), the y-axis describes the resulting amplitude as percentage of the mean values of the respective control condition. Black: placebo, blue: MPH; filled squares: single-pulse responses, open squares: double-pulse responses (ISI 3 ms).

In Fig. 3, the course of the relative amplitudes of unconditioned and conditioned MEPs is presented for control, go and nogo trials.

Analyzing the normalized data of the control condition, the only effect of statistical significance concerned the factor Pulse (F(1,10) = 155.4; p < 0.001) indicating that unconditioned MEPs were larger than conditioned MEPs (see Fig. 3, upper trace). The factor Pulse was also significant for go-left, go-right and nogo trials (F(1,10) > 16.86, p < 0.005).

In go-left trials (i.e., reaction with the left hand; MEP recorded at the right hand), a quadratic effect for the factor latency (T-quad(1,10) = 19.99; p < 0.005) was obtained. MEPs were larger at an interval between S2 and the TMS-pulse of 230 ms than at the other latencies under study.

### Nogo trials

**Figure 4:**
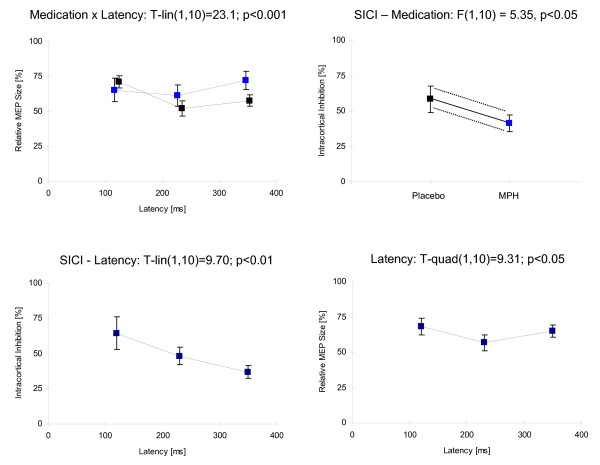
**Statistical effects in nogo-trials**. Means ± SE are shown. Left-top: Highly significant medication × latency interaction effect. Under methylphenidate (MPH, blue squares), MEP amplitudes increase in the time range analyzed. In the placebo condition (black squares), the opposite behaviour can be seen. Right-top: Under MPH, short-interval intracortical inhibition (SICI) is smaller than in the placebo condition. Left-bottom: SICI decreases linearly from 120 ms to 350 ms. Right-bottom: MEP amplitudes show a quadratic behaviour. They are smallest at latency 230 ms.

Fig. 4 illustrates the significant statistical effects obtained for nogo trials.

A highly significant linear effect resulted for the Med × Latency interaction (T-lin(1,10) = 23.05; p < 0.001): Under MPH, MEP amplitudes increased with time whereas, under placebo, they decreased (see Fig. 4a).

A specific test (post-hoc analysis) comparing the go-right vs. nogo-right trials at the TMS latency of 120 ms revealed a significant decrease of relative MEP amplitudes only for MPH (48.2 ± 56.0%; F(1,10) = 6.41, p < 0.05) but not for placebo (10.7 ± 42.8%; F(1,10) = 0.79, n.s.) indicating an earlier inhibitory effect for MPH.

The Med × Pulse interaction also turned out to be significant (F(1,10) = 5.74; p < 0.05). Accordingly, a medication effect was obtained when considering the SICI, i.e., the ratio of conditioned and unconditioned MEPs (F(1,10) = 5.35; p < 0.05): SICI was stronger under MPH (MPH: 41.3 ± 20.0%; placebo: 58.5 ± 31.2%; see Fig. 4b).

Besides the effects concerning medication, other TMS stimulus- or task-related effects were observed in nogo trials. MEP amplitudes were higher when inhibiting responses of the right hand compared to the left hand (nogo-left: 52.8 ± 8.0%; nogo-right: 73.8 ± 26.3%; factor Side: F(1,10) = 11.54; p < 0.01).

For the Latency × Pulse interaction, a highly significant trend was obtained: With latency between S2 and TMS pulse increasing, the difference between the amplitudes of single-pulse and double-pulse MEPs increased in a linear fashion (T-lin(1,10) = 20.69; p < 0.001). Accordingly, a Latency effect was obtained for the SICI (T-lin(1,10) = 9.70; p < 0.01; see Fig. 4c).

Compared to the other two latencies being measured (120 ms, 350 ms), the MEPs were significantly smaller at the 230 ms latency (factor Latency: T-quad(1,10) = 9.31; p < 0.05; see Fig. 4d).

## Discussion

In previous TMS studies, the effects of MPH on motor system excitability were investigated in a resting condition. To our knowledge, this is the first study dealing with MPH effects on motor system excitability in a response inhibition task requiring volitional inhibition and execution of prepared motor responses. Using this approach, a distinct pattern of excitatory and inhibitory effects could be revealed.

### Task paradigm

We used a S1-S2 paradigm and introduced a reward system with a challenging time frame of 350 ms for the response. The rather small standard deviations of the subjects' mean reaction time and low number of impulsivity errors (see Table 1) clearly indicate that all subjects tried to respond as fast and accurately as possible in order to maximize their profit. Thus, the reward system provided for well-defined test conditions. Otherwise, e.g., the MPH effect on reaction time could not have been detected. Additionally, it seems likely that the effort to inhibit the prepared response were higher due to the reward system [22].

### Methodological aspects

TMS pulses were applied at 120 ms, 230 ms or 350 ms after the S2 stimulus. These values were chosen according to the N1, N2 and P300 components of event-related potentials, which are related to inhibitory processes [23]. Of course, three different latencies provide only limited information about temporal characteristics. But nevertheless, we could reveal a distinct pattern of excitatory and inhibitory effects over time.

In nogo trials, SICI increased over the 350 ms latency range. Thus, our results are in line with the study of Sohn et al. indicating that SICI is actively involved in volitional inhibition [21].

MEPs (recorded at the right m. abductor digiti minimi) in nogo-right trials were larger than in nogo-left trials. A selective activation in relation to the prepared response (including spinal mechanisms) could have accounted for this effect in our two-sided experiment [24,25]. But no significant interaction effects containing the factor 'Side' were obtained in the nogo condition. This finding indicates that inhibitory effects occurred bilaterally. Neuroimaging studies have shown selective cortical activation related to volitional inhibition in the prefrontal cortex and supplementary motor cortex. Bilateral activation of these areas may explain non-selective inhibition occurring in nogo trials [21].

Over-all excitability and SICI developed differentially over the latency range analyzed. Whereas linear effects were observed for SICI, over-all excitability showed a quadratic time course. This relation suggests that there is an interplay of different excitatory and inhibitory mechanisms being involved in the execution and inhibition of a prepared response [1].

### MPH effects

The main objective of this study was to investigate the MPH effects on motor system excitability and motor control, respectively.

Subjects reacted faster under MPH but not at the cost of performance errors. Quite the contrary, there was a trend towards fewer impulsivity errors under MPH. These effects at the performance level may indicate improved motor functions under stimulants as it had already been stated by Rapoport et al. nearly 30 years ago [26].

For SICI, a condition-specific effect was found. Whereas no significant effects were obtained in control and go trials, SICI was significantly enhanced in nogo trials under MPH (compared to placebo).

Besides this SICI increase, an earlier inhibitory effect (at 120 ms) and an increase of MEP amplitudes over time under MPH were observed in nogo trials. So, effects on motor system excitability during response inhibition were of excitatory and inhibitory nature.

Relating this pattern of TMS effects to the functioning of a car, it is not just the gas or the brake pedals which work better. The effects of a single dose of MPH (20 mg) in healthy adults may be best described as a car with a better tuning.

### Limitations of the study

Two left-handed subjects were included in the small sample which may have affected the results. However, the same result pattern was obtained when analyzing the data without the two left-handed subjects.

For data analysis, some assumptions were made (e.g., upper and lower MEP limits) which may have biased the results. On the other hand, it seems unlikely that these assumptions affected the placebo and the MPH results differentially.

## Conclusion

TMS allows to study dynamics of motor system excitability in response inhibition tasks, reflecting a pattern of excitatory and inhibitory effects. This pattern is indicated to be significantly altered by MPH in healthy adults. Thus, this neurophysiological approach could extend the spectrum of neurophysiological methods (in addition to event-related potentials and functional neuroimaging [23,27]) to investigate response inhibition, e.g., in children and adults with ADHD.

## Abbreviations

ADHD: attention deficit hyperactivity disorder; EMG: electromyography; ICF: intracortical facilitation; ISI: interstimulus interval; MEP: motor evoked potential; MPH: methylphenidate; MT: motor threshold; RMT: Resting motor threshold; SICI: short-interval intracortical inhibition; TMS: transcranial magnetic stimulation.

## Competing interests

The authors declare that they have no competing interests.

## Authors' contributions

OK contributed to the conception of the study, continuously supervised the study and drafted the manuscript. MD contributed to the design of the study, was responsible for data collection and helped to draft the manuscript. PS was involved in the collection of the data, in their analysis and interpretation. WG, JB and GM contributed to the conception of the study and critically revised the manuscript. HH conceived the study, was responsible for data analysis and helped to draft the manuscript. All authors read and approved the final manuscript.
